# Co-producing sexual health services with minoritised communities: learning from a longitudinal qualitative evaluation

**DOI:** 10.1136/bmjph-2025-003335

**Published:** 2026-06-09

**Authors:** Fiona Fox, Aisha Namurach, Peninah Achieng-Kindberg, Lindsey Harryman, Heidi Andrews, Matthew Wilson, Nathan Speare, Temilola Adeniyi, Marsha Doran, David Dravie-John, Momin Mohamed, Jeremy Horwood

**Affiliations:** 1 The National Institute for Health and Care Research Applied Research Collaboration South West, Brsitol, UK; 2 Population Health Sciences, Bristol Medical School, University of Bristol, Brsitol, UK; 3 Public Contributor, Cardiff, UK; 4 African Voices Forum, Bristol, UK; 5 University Hospitals Bristol and Weston NHS Foundation Trust, Bristol, UK; 6 Bristol Health Partners Academic Health Science Centre, Bristol, UK; 7 The Brigstowe Project, Bristol, UK; 8 University of Bristol, Bristol, UK; 9 NIHR Health Protection Research Unit (HPRU) in Evaluation and Behavioural Sciences, University of Bristol, Bristol, UK

**Keywords:** Community Health, Qualitative Research, Sexual Health, HIV, Community Participation

## Abstract

**Introduction:**

Co-production is an important practice for addressing health disparities and developing equitable services with underserved communities. Co-production aims to ensure that underserved communities are central to the design of services that reflect community needs. African and Caribbean heritage communities (ACHC) face heightened risks of HIV due to stigma, discrimination, social, economic and structural factors, leading to significant health inequities. Despite a national target to end new HIV transmissions by 2030, HIV remains prevalent. Common Ambition Bristol (CAB) is a co-production project aiming to increase HIV knowledge and testing. CAB’s Project Delivery Group (PDG) involves ACHC community members and sexual health staff working in equal partnership to improve sexual health services for ACHC.

**Methods:**

A longitudinal qualitative evaluation explored the process of CAB’s co-production over time. Interviews were conducted with members of CAB’s PDG at three time points. Interviews examined PDG views and experiences of the co-production process. Data were analysed thematically.

**Results:**

Five themes are reported which relate to equitable power-sharing and inclusive decision-making: (1) Acknowledging power imbalances and negotiating roles, (2) Appreciating commonalities and the importance of language, (3) Benefits of sharing lived and sexual health experiences, (4) Negotiating different opinions safely and (5) Co-production: the messy middle.

**Conclusions:**

Findings underscore the potential of co-production to drive meaningful progress in health equity. Key elements of co-production which promote equitable power-sharing and effective decision-making are: (1) acknowledging and addressing unequal power structures which may affect group dynamics; (2) fostering reciprocal learning from lived experience and sexual health expertise; (3) recognising and appreciating personal and sexual health commonalities; (4) negotiating and developing ways to ensure equitable decision-making and (5) agreeing a shared language which reflects 1–4.

**WHAT IS ALREADY KNOWN ON THIS TOPIC**

- Co-production is crucial to tackle health inequities and increase access to services for underserved communities.
- Achieving equal power sharing and inclusive decision-making between health service providers and service users is challenging and historical disparities exacerbate this problem.
- Longitudinal insights are needed to better understand the potential challenges and facilitators when co-producing health services with minorised communities.

**WHAT THIS STUDY ADDS**

- This longitudinal evaluation highlights that when co-producing service improvement with racially minoritised communities:
  - reciprocity, equal power sharing and inclusive decision-making require continuous attention and re-negotiation.
  - acknowledging and understanding the inherent, historical power structures which impact on the lived experience of minoritised communities is essential. This includes personal reflection on degree of privilege and influence and consideration of how this affects group dynamics.
  - Equal attention should be paid to the value of lived and sexual health experience, with a shared language to reflect this.
  - Opportunities should be made to enhance transparency and personal connection between all stakeholders within the project.

**HOW THIS STUDY MIGHT AFFECT RESEARCH, PRACTICE OR POLICY**

- This study demonstrates to service providers and policy-makers that effective co-production with racially minoritised communities requires sustained relationship-building, transparency and active attention to historical power imbalances to ensure equitable decision-making.
- It highlights the need to allocate time, resources and structural support to embed genuine community leadership and create lasting, culturally responsive health services.
- Building evaluation into the co-production process will facilitate iterative reflection, allowing teams to address challenges as they emerge.
- Policy-makers should consider incorporating co-production as a standard requirement in the design and delivery of public health services, ensuring that minoritised communities have a voice in shaping policies and interventions that affect their lives. The Common Ambition Bristol toolkit guides service providers who want to use co-production with minoritised communities to improve health services.

## Background

 Co-production is increasingly recognised as a key practice for addressing health disparities and developing equitable services with and for underserved communities. It has been described as, ‘*imperative to inclusive and evidence-based service development’*.^1^ Co-production involves an equal working partnership between people who use health services and service providers, in a mutual effort to improve access to services. This requires engaging groups of people at the earliest stages of service design, development and evaluation. It involves sharing power and decision-making throughout the process of change. This increases the likelihood that services which lack diversity are adapted to be acceptable and meet the needs of the communities they serve.^2 3^ Co-production initiatives like the Act Early programme, addressing health inequities to improve children’s health, have been successfully implemented using defined principles to ensure meaningful and sustainable engagement with communities.^4^

HIV is an area of significant health inequities. People from African and Caribbean heritage communities (ACHC) are disproportionately affected by high rates of HIV transmission, late diagnosis and limited access to preventive measures, such as HIV testing and PrEP (Pre-Exposure Prophylaxis—a tablet taken to reduce the risk of HIV).^5^ Recent UK National Institute for Health and Care Excellence^6^ guidance highlights the need to improve access to sexual health services and reduce barriers for underserved groups, including addressing stigma, ensuring accessible language and acceptable testing locations. The involvement of ACHC’s in service improvement is key to improving the uptake of HIV testing and PrEP.^6^ Guidance advocates co-producing interventions by engaging communities in planning, design, implementation and evaluation.^6^ However, the *c*hallenges of achieving effective co-production with underserved or minoritised communities have been documented.^7 8^ For example, creating participatory spaces does not immediately equate with equitable power sharing and inclusive decision-making.^9^ The importance of addressing and levelling hierarchical structures and some strategies to achieve this have been suggested.^10^,^12^

### Common Ambition Bristol

Bristol is considered a high HIV prevalence city and has a late diagnosis rate that is higher than average. Meeting the United Nations Programme on HIV/AIDS goal of eradicating HIV inequities and new HIV infections by 2030^13^ is a challenge for the city. In Bristol, a disproportionate number of people with undiagnosed HIV (17%) or who receive a late HIV diagnosis are from ACHC.^14^ In 2021, 20% of new HIV diagnoses in Bristol were among people of ACHC^13^ despite these groups making up only 5% of the city’s population.^15^ This population had lower attendance at the city’s sexual health clinics and lower uptake of HIV tests.^16^ This is associated with poor health outcomes and is a significant challenge for Bristol.

In 2019, Bristol became an HIV Fast Track City,^17^ a global initiative where groups plan, deliver and evaluate health services collaborating to meet the 2030 goal. This collaboration was awarded a Health Foundation grant for the Common Ambition Bristol (CAB) programme, which aims to tackle health inequalities by increasing HIV knowledge and testing with ACHC in Bristol. CAB’s model of co-production involves a Project Delivery Group (PDG, see table 1). This is made up of six ACH members of the public working in equal partnership with four sexual healthcare staff (from both the National Health Service (NHS) and Voluntary, Community and Social Enterprise (VSCE)). PDG membership has changed over the 3 years but on average 70% of the PDG are of ACH. Together, the PDG co-produced interventions to tackle HIV stigma and increase HIV testing and access to PrEP. These include community outreach, dedicated testing clinics and targeted health promotion events and resources. The community members (CMs) were paid for their time.

**Table 1:** Common Ambition Bristol (CAB) co-production structure

Group	Abbreviation	Role	Membership
Project Delivery Group	PDG	Co-produce and deliver interventions to meet CAB aims	CM: ACH community members (6) SHS: Sexual health staff (4) Project coordinator
Project Advisory Group	PAG	Provide advice, governance and implementation support to PDG	Representatives from all partner organisations: VSCE Community organisation Healthcare Trust/service provider City council University

ACH, African and Caribbean heritage; CM, community member; SHS, sexual health staff; VSCE, Voluntary, Community and Social Enterprise.

CAB is led by Brigstowe, a charity for people living with HIV. Project partners include: African Voices Forum (an umbrella organisation for local community groups in Bristol), Bristol City Council Public Health, Bristol’s NHS sexual health provider and the University of Bristol. Representatives from these organisations formed a Project Advisory Group (PAG, see table 1). This group provided advice, governance, implementation support and risk management for CAB. In contrast to PDG, 30% of the PAG are of ACH.

An evaluation of the CAB co-production process was built into the project from the outset, led by the University of Bristol. CAB provided a unique opportunity to better understand the process of co-production over an extended time period (3 years). This work is needed to highlight key challenges and facilitators to achieving effective co-production with minoritised communities.

## Methods

The aim was to undertake a qualitative evaluation of the co-production process, in order to (1) provide iterative anonymous feedback to the PDG to enhance working relationships and (2) understand how the PDG shared responsibility, decision-making and power over the 3 years. Three overarching concepts implicit to effective co-production provided a structure to evaluation. These were selected via a process of reviewing and amalgamating key principles for conducting co-production^18^ and domains for evaluating co-production^19^ (see table 2). These three key domains (see column 1 table 2) were used to develop the interview topic guide.

**Table 2:** Principles for conducting and evaluating co-production

Key domains for analysis	Co-production principles	Co-production evaluation domains
Current study	NIHR 2021^18^	Eisenstadt 2015^19^
Power sharing and relationship building	Sharing of power–the research is jointly owned and people work together to achieve a joint understanding Building and maintaining relationships–an emphasis on relationships is key to sharing power	More focused on project outputs/interventions than process of co-production
Role negotiation and inclusive decision-making	Including all perspectives and skills–make sure the research team includes all those who can make a contribution Respecting and valuing the knowledge of all those working together on the research–everyone is of equal importance	Degree to which decision-making is participatory Degree to which roles are dispersed/shared
Reciprocity and personal development	Reciprocity–everybody benefits from working together	Degree of mutuality/reciprocity Human capacities developed Assets created/uncovered

NIHR, National Institute for Health and Care Research.

A qualitative design explored how the CAB co-production working model functioned in practice. Longitudinal interviews with PDG members were conducted annually over the 3 years.

### Recruitment and consent

In year 1, researchers JH and FF discussed the evaluation methods with the PDG. Subsequently, all PDG members were given an information sheet and provided written consent. Prior to each interview, FF reminded them of the consent process and took verbal consent.

### Semistructured interviews

One-to-one interviews were conducted by FF via MS Teams or phone, according to preference. Topic guides structured the interviews, with questions derived from three key domains (see column 1, table 2). Each year, additional questions focused on specific activities relevant to the stage of the CAB project (eg, joining the PDG, delivering interventions). All interviews were audio-recorded and transcribed verbatim by a professional company.

### Analysis

The analysis of the interview data followed a process of inductive thematic analysis.^20^ All interview transcripts were anonymised before analysis. FF read through all transcripts and survey responses and coded the data inductively, using NVivo V.12 software to assign codes to segments of text. A subset of transcripts was double-coded (JH) to increase reliability and key themes were agreed. Findings were then shared in two ways: (1) iterative feedback was shared with the project co-ordinator, PDG and PAG via a table of issues affecting working practice. Plans to address these were then agreed and documented by PDG and (2) The inductively derived themes were written up and are reported below. To ensure anonymity, certain identifying details are removed from quotes and square brackets [] are used.

### Patient and public involvement

The CAB evaluation team worked in partnership with members of ACHCs. Members of the PDG were actively involved in shaping decisions about the design and delivery of this evaluation. Four members of the PDG and two community researchers are co-authors. Two members of African Voices Forum helped interpret the findings and are co-authors on this paper.

## Findings

### Sample

Membership of the PDG changed over the 3 years, with three people leaving and new members joining. The following terms: CM and SHS (sexual health staff) are used with agreement from the CAB team. In year 1, 10 interviews were conducted (5 CMs/5 SHS); in year 2, 11 (5 CMs/6 SHS); and in year 3, 6 (3 CMs/3 SHS). Every member of the PDG took part in at least one interview, with two taking part in all three. Of all the PDG members who participated in the evaluation, five were male and nine were female. The CMs were of African or Caribbean heritage and had a range of professions. SHS included roles such as health advisors and nurses.

The following themes are reported: (1) Acknowledging power imbalances and negotiating roles, (2) Appreciating commonalities and the importance of language, (3) Benefits of sharing lived and sexual health experiences, (4) Negotiating different opinions safely and (5). Co-production: the messy middle.

### Acknowledging power imbalances and negotiating roles

In year 1, SHS expressed some uncertainties regarding their role within the PDG, based on their white British heritage and status as healthcare staff. Initially, this seemed to hinder their confidence in building effective working relationships with the CMs, who were all of ACH. Doubt about their use of language during PDG meetings was raised but this changed within the first year, as they felt more confident to adopt the CMs’ use of language. For example, one SHS reported:

I definitely felt more comfortable saying the words ‘black’ and ‘white’ since I joined this project whereas (before) I felt like ‘oh I can’t say the word ‘black’, I can’t say the word ‘white*’* (SHS Year 1 interview).

The SHS reported their increased understanding of the inequities experienced by CMs within the healthcare system, for example when the group was developing a project logo for CAB:

They (CMs) felt that if you put the words ‘HIV’ and ‘black’ on a logo then that’s stigmatising black people to have HIV. That came across in the meeting and at that point I realised how I might … how people in healthcare might come across. And maybe that’s the reason we don’t have as much involvement with the black community, that maybe they don’t feel that they’re being treated with … the same free will and the same choices as a white person? (SHS Year 1 interview).

This deeper understanding of inequities led to impacts in their sexual health practice and organisations:

I think has probably both affected my sexual health practice … because just hearing more from a different part of the community about their experiences of sexual health, their opinions about how services should be delivered. Particularly thinking about how we talk about HIV risk amongst the African and Caribbean community in a way that isn’t adding stigma. There’s been times when I’ve gone back to [ORGANISATION] and said actually, I’m not sure that this wording is really helpful … I’ve felt more confident as well saying ‘this doesn’t feel comfortable, can we try rewording how we talk about this in a way that it’s still helpful information but doesn’t reinforce messages that HIV is an African problem’ (SHS, year 2 interview).

This recognition of historical, systemic inequalities affected the degree to which SHS spoke up or contributed to decision-making within the PDG. Some felt that in order to share power effectively, the white members of PDG needed to first ‘hand it over’:

because there’s been this kind of prevalence of white power, my feeling is that there is a need to almost relinquish control entirely and … before we share power, I think we need to give them power for a while. Otherwise, it’s not balanced (SHS, year 1 interview).

Consequently, in the first year, SHS tended to take a back seat in meetings, allowing CMs to lead decisions. Some SHS requested clarity over when or how they should provide more input, to understand, “*what would be helpful or empowering”*? (SHS year 1 interview). However, by year 2 the combination of respect afforded by SHS and their increasing confidence was noted by the CMs:

They’ve (SHS) always been respectful and always said that the final decision regarding interventions and stuff remains with us (CM’s) … but I think they (SHS) have now become comfortable to giving their own ideas as well and are very respectful where some might not identify as African or Caribbean, but are very aware there’s a disparity going on and they’re very respectful in terms of that (CM, year 2 interview).

### Appreciating commonalities and the importance of language

It was raised in year 2 that language used to describe PDG members could reinforce unequal power structures. The term ‘sexual health professionals’ was frequently shortened to ‘professionals’ which some felt disregarded the varied professional backgrounds and expertise of CMs.

Let’s not refer to sexual health professionals—we are all professionals here (CM, year 2 interview).

In one meeting, a change in language was proposed, to better reflect the expertise of all PDG members. It was decided that CMs were to be ‘experts in lived experience’ and SHS ‘experts in sexual health’. It was agreed this language contributed to a more balanced and respectful working environment.

Appreciating each other’s areas of professional expertise was important and highlighted commonalities. In the first year, all meetings were conducted online. When COVID-19 pandemic restrictions were lifted, the PDG was able to meet in person and get to know each other better. At this point, they discovered some shared experiences, such as job roles, which reinforced camaraderie:

Because we were all people …. some of the community members are nurses, so I didn’t see them as a community member I just saw them as nurses … a fellow nurse (SHS year 1).

### Benefits of sharing lived and sexual health experiences

Sharing of lived and sexual health expertise was acknowledged to be reciprocal. CMs learnt more about sexual health via the CAB induction training and from SHS in meetings. Through this process, human capacities were developed. A CM explained their personal gains:

I think I know a few more people in the community. I know more about sexual health and HIV, so that’s improved my knowledge. I like the fact that I’m contributing as well to our community and also, as a [Job role], I can use that in my practice as well. Yes, I think I’ve gained confidence as well and communication, yes (CM, year 3 interview).

SHS learnt from the lived experience of CMs. By year 2, a white SHS commented on their increasing confidence when speaking up about issues affecting the ACH community:

I’m not necessarily coming from such an outsider position but I’m still careful to check in with the community members about what they feel … I suppose my understanding of what they might think has changed. So, perhaps I’ve learned lots about … how the community might feel about something and therefore I feel better equipped to offer something that feels more likely to be taken up by them, because it feels like I’ve heard them talk about this before, or something similar. So there’s something about awareness of their lived experiences that I’ve developed through working and co-creating together. So I feel more confident now (SHS, year 2 interview).

Over time, this integration of sexual health and personal lived experiences facilitated more equal decision-making. One CM explained that when CMs raised ideas, SHS drew on their experience of sexual health service delivery to explain how or why these would or wouldn’t work in practice:

Yes, it was equal because they (SHS) give their feedback, they give their input from their side of the job from their sexual health side and we as a community give our feedback and we did work as a team. It doesn’t ever feel like they were the professionals and we were … just community members. We just worked together which was quite good and people that didn’t have more knowledge about it (sexual health) would be able to get a bit more information and they gave us a bit more insight. (CM, Year 2 interview).

By year 3, it was felt that all voices were expressed and heard equally:

I think we’re (SHS) still aware of it in the sense of wanting to make sure that we don’t want to take the space but I think there’s less anxiety around that and I feel like there’s a comfort being able to say my opinion and know that actually it’s going to be heard amongst other voices rather than over or above those voices (SHS, year 3 interview).

### Negotiating different opinions safely

In years 1 and 2, the PDG engaged in a process of designing, developing and piloting interventions. It was largely agreed that decisions were made collaboratively via consensus. Mutuality and reciprocity were key to this process, as the group negotiated ways to achieve agreement. Some valued options to give feedback outside of meetings to the project co-ordinator. This enabled them to voice an opposing opinion or raise an issue that they had not felt able to express in the meeting.

Opportunities to work through differences in opinions during meetings were sometimes limited by time pressures. It was suggested facilitation could ensure it felt safe to discuss opposing views. However, examples were given of when differences were negotiated safely and successfully. These highlight the primacy given to achieving group consensus over individual preference:

We have differing views but by having a discussion, listening, questioning there is an outcome, and if it’s not the one you want, accepting that it is not personal, it is a win (CM, year 2 interview).

### Co-production: the messy middle

This theme illustrates unforeseen issues between the wider group of project partners (see table 1), which had a significant impact on the CAB co-production process.

#### From ‘behind closed doors’

The CAB PAG included representatives from all partner organisations and was designed to support the PDG (see table 1). Some PAG members held influential positions within sexual health service provision and commissioning. In years 1 and 2, the PAG and PDG had separate monthly meetings. CMs were invited to attend PAG, to represent the PDG and share information between the two groups. However, the timing of PAG meetings (during the working day) was prohibitive for some CMs. This led to a sense of disconnect between PDG and PAG, which was especially evident in year 1 interviews:

I don’t think we are connected as such and I don’t know if maybe for me, personally, because maybe the non-attendance at PAG perhaps so it’s not really linking properly. So, I feel it’s a bit disjointed there (CM, year 1 interview).

CMs who did attend sometimes struggled with actively participating in PAG meetings. However, over time, familiarity led to increased confidence:

I didn’t feel quite comfortable to talk strong at PAG which is why I didn’t attend PAG meetings. When I was asked to attend PAG I was like ‘no, you’re not seeing me there because all these health professionals around and that is a no from me. You’re going to be talking in your posh language and I’m not going to get it and I’m going to just look an idiot’ and then I attended and I thought ‘that’s not too bad’ and yes I just think, again, just like PDG I’ve just kind of built up a relationship that I’m alright to just talk*.* (CM year 2 interview).

This sense of disconnect contributed to uncertainties around decision-making within CA, an issue raised in all three rounds of interviews. Year 1 highlighted uncertainty about whether PDG or PAG makes final decisions about CAB. There was some associated unease that the majority of PAG members were white (70%). It was felt the lack of lived experience among this group may impact on decisions about interventions for the ACH community’.

It feels like that thing of gatekeeping and kind of ‘what are you (PAG) actually doing, are you resisting the potential for us (PDG) to make changes or for things to happen’ in a kind of holding the purse strings way? it’s a really tricky dynamic because it feels like actually all these professionals (PAG) not from the (ACH) community taking advisory decisions of the overarching project direction … I just think there’s a resistance between what the PAG are doing and what the PDG want (SHS, year 2 interview).

#### Opening a sore wound

Concerns about disconnect came to a head at the end of year 2, when the PAG and PDG participated in a joint workshop to review CAB’s progress and agree future plans. Following a productive working day, strong opinions were shared regarding the lived experiences of CMs and their views around the sustainability of funding of services for ACH communities:

If CAB does cease, it will really annoy me, it will because I will feel like it was a tick box exercise. That’s what I will feel and it will be that whole ‘we knew it, that’s why we weren’t really connecting with you because we knew you were just one of those many that come in, throw some money in to show we did try and then you go again’ so you become that statistic project. Even the word project annoys me … Because it’s like the project means that it’s not long-lasting isn’t it? If it’s a project, it’s not here to stay (CM, year 2 interview).

Another CM described this meeting as ‘opening up a sore wound’:

It was helpful but it also raises a few issues because I think when you’re working in the community and you know exactly the pitfalls and what’s required and you’re not getting it and then you feel like you have to justify a reason for having that service when it’s a right to have it. It felt like you have to give justification for them to have that service when nobody else needs to do that. So, that kind of opened up a sore wound really (CM, year 2 interview).

One of the SHS reflected on inherent issues of ‘power and privilege’ and contrasting work and lived experiences across PAG and PDG:

It’s quite interesting being in a room with people who you know are influential leaders in the sector that you work in … talking about things that are then very personal and obviously there were some moments that were quite emotional in the day and I guess there are some things about power and privilege within that experience where it’s not just about identity, but it’s also about the power you hold within your employment and also your socio-economic status … everyone is feeling a lot in those meetings but some of us probably leave and carry that feeling in a different way outside of the meeting than others and that’s something I wouldn’t have ever foreseen as an experience (SHS, year 3 interview).

#### Working together towards a common ambition

Following the joint workshop (5.2), the two groups (PDG and PAG) met separately to reflect and debrief on their experiences. It was agreed that both groups should come together in a facilitator-led, in-person workshop to share their reflections and suggest solutions for future working. At the workshop, the need for better transparency and personal interaction between PAG and PDG was prioritised. It was decided this could be achieved in several ways (1) monthly opportunities for PAG and PDG to meet in person between meetings and share food. Meetings were held in the evenings, at a central community location to facilitate attendance for all. (2) PAG members joined PDG meetings to share information about their roles and their organisations. It was widely agreed that this was a significant point, enriching the process of co-production within the whole CAB team:

There was probably some feelings that had been underlying for some time that came to a head in that time and I think probably needed to … the response to it has also been really important and I reflected afterwards that just the space and the opportunity to have an emotive discussion about the experiences of a marginalised part of our city, in the room with people who are part of the decision making that is around that subject felt like it was really important. A really an important moment (SHS, year 3 interview).

During interviews, both SHS and CMs reflected that following this facilitated workshop, the perceived balance of power between PAG and PDG shifted, via improved relationships and open dialogue:

Up until that point that PDG sort of were accountable to PAG and were answering to the PAG group and in that meeting it felt like actually, the PDG were holding PAG to account in some ways (SHS, year 2 interview).

One CM reflected on how the balance of power was negotiated between PAG and PDG over time. They highlighted how the working relationship between the whole CAB team demonstrates mutual commitment towards a common ambition:

I think obviously there was a bit of - I don’t even know how to describe it - back and forth between kind of what is beyond PDG and what’s being done behind closed doors and … I don’t think that’s a bad thing at all. I think we’ve reached a level of comfortability that as a marginalised group, it’s really hard to reach that comfortability to speak up and ask those questions. So, I think it’s a good sign …I don’t think there’s really much that we (PDG) said that PAG haven’t agreed with or have had a big input in. I know in terms of interventions and stuff like that we (PDG) … get the final say and it’s been really evident when we have the team meetings which are together and PAG members get stuck in with brainstorming some interventions … So, yes, not only do they say it, they show it as well (CM year 3 interview).

## Discussion

The current findings based on longitudinal qualitative data offer unique insight into challenges and facilitators for co-production when racially minoritised CMs work with health services staff. They indicate that within this context, key elements of co-production which promote equitable power-sharing and effective decision-making are (1) acknowledging unequal power structures which may affect group dynamics, (2) reciprocal learning from lived experience and sexual health expertise, (3) recognising and appreciating personal and sexual health commonalities, (4) negotiating multiple ways to ensure equitable decision-making and (5) agreeing a shared language which reflects 1–4. In addition, the findings reflect how all CAB partners navigated emotional challenges and vulnerability, which were central to the development and maintenance of trusting relationships.

The findings highlight that when co-producing services, power sharing and participatory decision-making has relevance beyond the immediate working/delivery group (PDG), to the wider team of stakeholders who may be involved in advising the project (PAG). While originally focusing the evaluation on dynamics within the PDG, the findings reveal the significant impact of these wider relationships with partners involved in the co-production process. This demonstrates the importance of transparency, so that decision-making is not seen to be taking place ‘behind closed doors’.

### Comparison with existing literature

The current findings reflect that if lived experience of historical inequalities and inherent power imbalances are not adequately addressed, ‘sore wounds’ may be exposed within co-production working groups. It is crucial to allow space for these wounds to be named and heard. This requires the toleration of discomfort, and for service providers to acknowledge their position of influence and power. This ‘vulnerability among all stakeholders’ is echoed in other work, where researchers doing co-production reported feeling less in control of the process than usual.^21^

Various models of power-sharing are shaped by the cultural practices and historical contexts of the communities involved. This study worked with ACH communities, whose distinct experiences affect how they engage with services. For instance, cultural stigma surrounding sexual health and HIV and previous negative experiences of healthcare may influence levels of engagement with HIV testing.^22^ Acknowledging and discussing these cultural and contextual considerations is essential to ensure meaningful participation and address service development needs effectively.

Efforts to ‘hand over power’ may be a first step towards redressing imbalance. In the current study, SHS stepped back so CMs could initially take the lead in decision-making. This was echoed in a study where researchers *‘used the phrase ‘sit back’ to ensure that public-participants had their say’*.^23^ In the current study SHS wanted to ‘*hand over power first’* before sharing it, to address historical power imbalances experienced by people of ACH.

The negotiation of roles in decision-making (eg, about intervention delivery) may need attention depending on the group. As reported elsewhere this process may require ‘deconstruction of hierarchies’.^23^ In the current study SHS negotiated how and when to share their service delivery experience, so that it was useful or empowering for the PDG. Over time, the CMs developed their sexual health expertise and SHS gained deeper insight into the lived experience of people of ACH. This process has been described as the ‘*merging of experiential and evidence perspectives’*.^23^

Evidence suggests that successful co-production relies on a collaborative partnership where knowledge and resources are shared in a mutually beneficial way.^11^ Within the current study, the PDG agreed on language which emphasised the complementary value of their varying forms of expertise (eg, people with lived experience/sexual health expertise). This promoted a gradual reconstruction of roles within the PDG, facilitating more equal power structures and participatory decision-making. Benefits of this were both personal and professional. CMs reported learning more about sexual health. SHS noted increased confidence in suggesting changes to sexual health services. Farr conceptualises a similar generation of power which can instigate forms of social change.^24^

The ongoing work of building relationships is essential to promote effective co-production and clarifying roles is key to this.^25^ The current study extends this to wider project partners (eg, PAG). It was crucial for the CMs to understand the roles of PAG members, the organisations they represent and their degree of influence in decision-making within service improvement (eg, commissioning and/or service delivery). As shown in previous work, a focus on flexibility within relationship building is essential.^8^ The current findings suggest that spending time together, within and outside of meetings, can help increase personal connections and reinforce professional commonalitie*s*. The importance of meeting in a community location and in after-work hours is advocated by other studies.^25^ Evidence supports the current findings regarding the temporality and dynamics of trust, which requires continuous, collaborative efforts to promote confidence.^26^

### Strengths and limitations

The study benefits from a longitudinal design, evaluating the process of co-production over the course of 3 years and provides a nuanced understanding of the dynamics of collaboration over time. All interviews were conducted and analysed by FF. However, a subset of transcripts was double-coded (JH) to increase reliability. When taking consent, FF reminded participants about the confidential nature of interviews and the steps taken to ensure anonymity when analysing data and reporting findings. It is acknowledged that FF’s white ethnicity may have impacted on interview dynamics and the resulting data. A fuller discussion of this is presented below.

### Reflexivity/positionality

As a white researcher engaging with participants of ACH, FF’s social positioning was shaped by historical and contemporary power dynamics that structure racialised interactions. This positionality inevitably influenced how questions were asked, how responses were interpreted, and how participants chose to present their experiences. Throughout the research process, FF remained attentive to the ways in which her racial identity could shape rapport, trust, and disclosure, and approached interviews with a commitment to reflexivity and openness. By explicitly acknowledging these dynamics, FF aimed to minimise the reproduction of unequal power relations and to treat participants’ voices as authoritative accounts of their own lived realities. FF was also mindful of the additional complexity of her relationships with PDG participants, as a result of attending PAG and PDG meetings over the 3 years. This had the potential to shape both the dynamics of the interview and the ways in which accounts were shared or withheld. The findings were discussed with members of the wider co-production team who are of ACH to ensure the implications drawn out were appropriate.

## Conclusions

This study illuminates the complexities of co-production over 3 years. It provides examples of key challenges which arose and strategies adopted to address these. Findings show that when co-production teams work together to appreciate and learn from each other’s expertise, power dynamics can change over time. It also identifies the need for clarity over where decision-making actually lies. This highlights the importance of transparency and connection across all involved in the co-production process. When co-production teams involve members with lived experience of health inequities working alongside service providers and commissioners who influence service provision, it is crucial to continuously address inherent, historical power structures.

This study contributes to growing evidence on the importance of participatory approaches in service development. The CAB Toolkit^27^ based on the current findings provides a practical, detailed guide to co-producing health services with minoritised communities.

Future research could explore how co-produced services influence healthcare access, service uptake and health outcomes, particularly in minoritised communities. Studies could explore the role of healthcare organisations, policy-makers and funding structures in sustaining co-production efforts. This may ensure participatory decision-making is embedded into mainstream service development. Additionally, studies could explore the knowledge, skills, and attitudes needed for successful co-production. The role of intersectionality in shaping co-production processes would also be a fruitful area of future study. Such work will continue to address health inequities and co-produce more accessible services for minoritised communities.

### Recommendations

The table of recommendations (box 1) reflects insights arising from the specific study setting. They should therefore be interpreted as context-dependent, rather than universally prescriptive. While the current findings align with wider evidence on co-production, alternative approaches may be required in different organisational or community contexts.

Box 1 **Implications/recommendations**

**Power sharing**

- Awareness of historical unequal power structures—*Ask: what does this feel like/how does this apply to me/my service?*
- Handover power first to redress imbalance—give space for community voices and lived experience to be shared. *This may mean sitting back/agreeing when sexual health expertise is welcome*
- Sit with discomfort of role ambiguity—*This may mean feeling redundant for a while*
- Build relationships*—via transparency and regular in-person connection*

**Role clarity**

- Acknowledge power associated with role*—professional status eg. Service provider/commissioner*
- Value lived experience/listen to community voices—*give time and space for this*
- Name discomfort—*speak up!*
- Negotiate ways to share expertise—*ensure it is empowering and helpful*
- Jointly agree titles/labels which reflect expertise
- Acknowledge impact of unique positions*—‘insiders and outsiders’ of the community and services*

**Participatory decision-making**

May be facilitated by:

- Power sharing and role clarity as addressed above
- Give sexual health and lived experience equal value—negotiate ways to incorporate both
- Offer multiple ways to contribute opinions inside and outside of group meetings
- Find safe ways to express diverse opinions—facilitation may support this
- Allow time and space to work through differences in opinion—*facilitation may support this*

**Reciprocity/exchange**

- Notice and celebrate personal and professional benefits—*often unforeseen*
- Personal development may include:
  - gaining subject-specific knowledge
  - becoming more familiar and integrated with the community you are working with
  - learning more about structural inequalities
  - gaining confidence and taking this forward into relevant service or place of work

For a practical guide to co-producing health and care services, read the CAB toolkit.^27^

## Data Availability

All data relevant to the study are included in the article. Data are available on reasonable request.
